# Novel Insights into Hb Shaare Zedek Associated with β^0^-Thalassemia: Molecular Characteristics, Genetic Origin and Diagnostic Approaches

**DOI:** 10.3390/ijms25168578

**Published:** 2024-08-06

**Authors:** Surada Satthakarn, Wibhasiri Srisuwan, Naowarat Kunyanone, Sitthichai Panyasai

**Affiliations:** 1 Faculty of Allied Health Sciences, Burapha University, Chonburi 20131, Thailand; surada.sa@buu.ac.th; 2 Department of Medical Technology, School of Allied Health Sciences, University of Phayao, Phayao 56000, Thailand; 3 Department of Medical Technology, Chiang Rai Prachanukroh Hospital, Chiang Rai 57000, Thailand

**Keywords:** α-thalassemia, β-thalassemia, hemoglobin Shaare Zedek, Premier Resolution HPLC, genetic origin, α-globin haplotype, hemoglobinopathy

## Abstract

Hemoglobin Shaare Zedek (Hb SZ) is a rare structural α-Hb variant. Characterizing its genotype–phenotype relationship and genetic origin enhances diagnostic and clinical management insights. We studied a proband and six family members using high-performance liquid chromatography (HPLC), capillary electrophoresis (CE), PCR, and sequencing to analyze α- and β-globin genes and α-globin haplotypes. Pathogenicity predictions and a rapid diagnostic method were developed. The proband, his father, grandfather, and aunt had Hb migrating to the HbH-zone on CE and elevated fetal hemoglobin (HbF) on HPLC. Direct sequencing identified an A to G mutation at codon 56 of the α2-globin gene, characteristic of Hb SZ. Additionally, the proband carried a β-globin gene mutation [HBB.52A>T]. Mild thalassemia-like changes were observed in the proband, whereas individuals with only the Hb SZ variant did not exhibit these changes. Pathogenicity predictions indicated that Hb SZ is benign. The variant can be identified using restriction fragment length polymorphism (RFLP) and allele-specific PCR. The Thai variant of Hb SZ is associated with the haplotype [- - M - - - -]. Hb SZ is a non-pathological variant that minimally affects red blood cell parameters, even when it coexists with β^0^-thalassemia. HPLC and CE systems cannot distinguish it from other Hbs, necessitating DNA analysis for accurate diagnosis.

## 1. Introduction

Hemoglobin (Hb) variants, which are monogenic disorders, contribute to global health issues [1,2]. Effective screening programs are now routine, leading to a significant increase in the detection of abnormal hemoglobin across diverse populations. Consequently, the characterization of these variants has expanded, with approximately 1800 documented and archived in databases such as the “HbVar database” [3] and the “ITHANET database” [4]. While most variants are clinically insignificant, some can affect hemoglobin chain production and stability, potentially leading to hemolytic anemia. More severe phenotypes can emerge when α-globin and/or β-globin gene mutations co-exist, necessitating precise diagnosis.

The most common gene alterations contributing to the instability of the Hb molecule are substitutions that typically disrupt interactions between subunits and the heme pocket [5]. Studies have identified around 70 non-deletional mutations that can either destabilize globin chains or affect mRNA stability [3]. Therefore, accurate identification and differential diagnosis of clinically relevant Hb variants from non-pathological ones is crucial. Routine Hb electrophoresis or HPLC analysis often fails to diagnose many Hb variants due to their similar electrophoretic mobilities and retention times. Consequently, further investigation, primarily through DNA analysis, is often required to accurately characterize these unknown variants.

In Thailand, there are over 70 known structural Hb variants, primarily caused by mutations in the β- and α-globin genes [6,7,8]. The country’s national prevention and control program for hemoglobinopathies has facilitated the ongoing discovery of new variants. Thailand has a high prevalence of α- and β-thalassemia, as well as significant rates of the pathological structural variants HbE and Hb Constant Spring [9]. This co-inheritance of Hb variants with α- or β-thalassemia leads to complex thalassemia syndromes with varying clinical presentations.

Hb SZ is a rare structural α-Hb chain variant first identified in a Jewish family of Persian descent [10,11] and later in a Chinese patient [12]. However, the impact of the amino acid substitution on the structure and function of the Hb tetramer remains unclear. This study describes the first case of Thai patients with Hb Shaare Zedek (Hb SZ) in both the heterozygote form and in combination with β^0^-thalassemia. Initial screening using capillary electrophoresis (CE) and high-performance liquid chromatography (HPLC) was inconclusive due to similar migration patterns with the variants. This study aimed to investigate the genotype–phenotype relationship, pathogenicity, and genetic origin of Hb SZ to enhance our understanding of this rare structural variant.

## 2. Results

### 2.1. Hematological and Hb Analyses

The proband, a 5-year-old boy, presented with moderate anemia. This was characterized by significantly decreased mean corpuscular volume (MCV) and mean corpuscular hemoglobin (MCH) values. His mother exhibited similar findings. In contrast, his father showed no signs of anemia or altered red blood cell parameters. Similarly, low Hb and hematocrit (Hct) levels were observed in his paternal aunt (aunt-1). However, none of the other paternal relatives, including the grandfather, grandmother, and another aunt (aunt-2), exhibited anemia or any changes in red cell parameters (see Table 1). The microcytic, hypochromic red blood cells observed in the proband and his mother suggest a possible influence of thalassemia.

**Figure 1:**
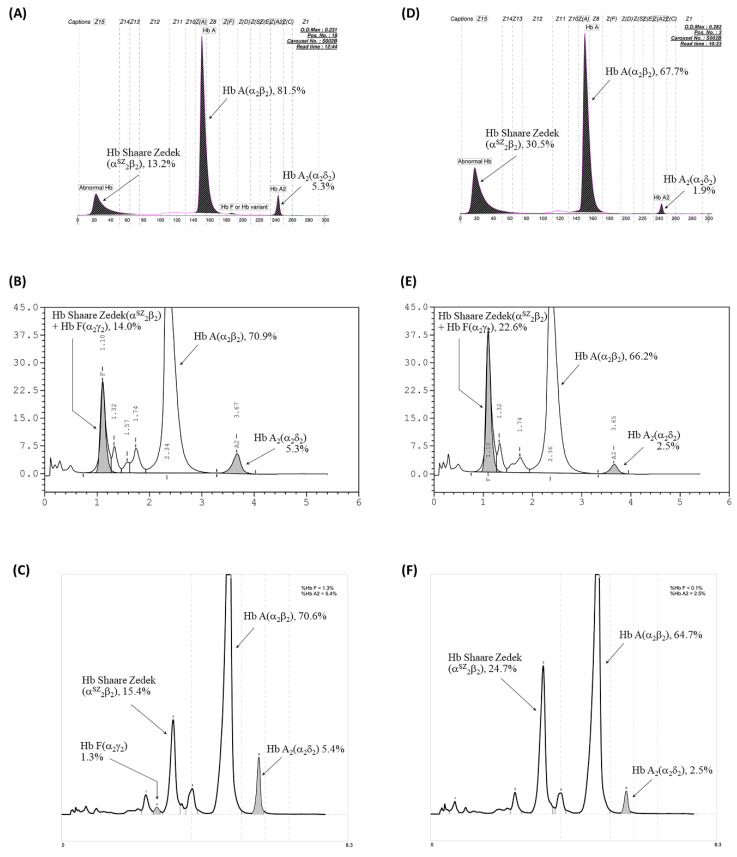
Hb analysis of the proband with Hb Shaare Zedek (Hb SZ) co-inherited with β^0^-thalassemia (**A**–**C**) and his father with heterozygous Hb SZ (**D**–**F**). (**A**,**D**) Analysis using the high-performance liquid chromatography (HPLC) VARIANT II system. (**B**,**E**) Analysis from the Premier Resolution HPLC system. (**C**,**F**) Analysis from the capillary electrophoresis (CE) system.

**Figure 2:**
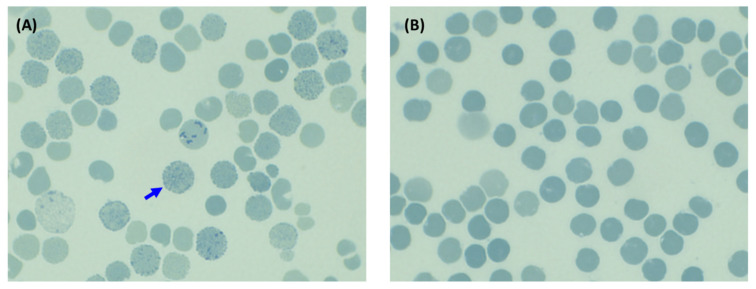
Red blood cell inclusion bodies induced after incubation with 1% brilliant cresyl blue for 1 h. The blue arrow points to red blood cells containing spherical inclusion bodies stained blue. (**A**) A positive control sample from a patient with HbH disease. (**B**) The proband sample.

Hemoglobin analysis using CE identified HbA, HbA2, and an unusual hemoglobin fraction migrating in zone 15 of the electropherogram, resembling HbH. This unidentified Hb was present in both the proband and his father, accounting for 13.2% and 30.5% of their total hemoglobin, respectively (Figure 1A,D), but was absent in the mother. The HbA2 levels of the proband and his mother were 5.8% and 5.3% of total Hb, respectively, which is consistent with the range typically seen in individuals with the β-thalassemia trait. In contrast, the father’s HbA2 level was slightly decreased (1.9% of total Hb). Based on the elevated HbA2 levels, an initial diagnosis of HbH disease co-inherited with the β-thalassemia trait was considered for the proband. The father’s findings suggested a possible simple HbH disease. Notably, no HbH inclusion bodies were detected in the red blood cells of either the proband or the father (Figure 2). VARIANT II-HPLC analysis revealed fractions of HbA, HbA2, and HbF. The HbF levels were elevated, constituting 14.0% and 22.6% of the total Hb in the proband and father, respectively (Figure 1B,E). These findings suggested a possible initial diagnosis of either β-thalassemia disease or hereditary persistence of fetal Hb (HPFH). Given the discrepancies between the CE and HPLC results, the possibility of an abnormal hemoglobin variant was considered.

Premier Resolution HPLC analysis of Hb revealed HbA, HbA2, and a prominent peak of an unidentified Hb fraction. This fraction eluted completely at retention times of 3.245 and 3.278 min for the proband and father, respectively, accounting for approximately 15.4% and 24.7% of their total Hb (Figure 1C,F). Similar results were observed in the grandfather and aunt-1, with the unidentified hemoglobin fraction eluting at slightly earlier retention times of 3.148 and 3.163 min using Premier Resolution HPLC. A comprehensive overview of the Hb analysis profiles and individual Hb fraction levels for the proband and his family members is presented in Table 1.

### 2.2. Globin Gene Analysis

**Figure 3:**
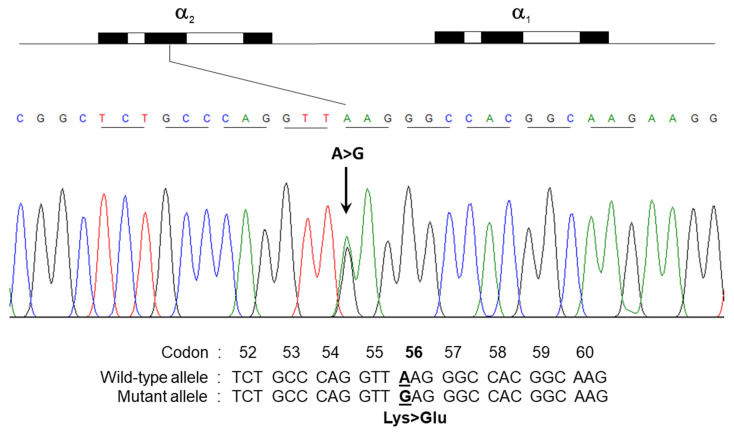
Mutational analysis of the HBA1 and HBA2 genes using DNA sequencing with selective amplification. The electropherogram shows the AAG to GAG change at the first nucleotide of codon 56 in the α2-globin gene, which is associated with Hb SZ.

Direct sequencing of the α- and β-globin genes identified an A to G mutation at the first nucleotide of codon 56 in the α2-globin gene of the proband and three other family members with the unidentified hemoglobin. No abnormalities were observed in the α1-globin gene (Figure 3). This A to G mutation, which substitutes lysine for glutamic acid, is characteristic of Hb SZ (HBA2:c.169A>G). It has been previously reported in Israeli and Chinese populations, but not in Thai individuals [10,12]. Additionally, an A to T substitution at codon 17 in the β-globin gene, the most common β^0^-thalassemia mutation, was identified in both the proband and his mother. DNA analysis confirmed that the proband carries both the Hb SZ and β^0^-thalassemia mutations in a heterozygous state, indicating a previously unreported complex thalassemia syndrome. The father harbored only the Hb SZ (simple heterozygous) mutation, while the mother carried the β^0^-thalassemia mutation. Therefore, the proband inherited the Hb SZ mutation from the father and the β^0^-thalassemia mutation from the mother.

### 2.3. Confirmation of Mutated Hb SZ Variant by PCR-Restriction Fragment Length Polymorphism (RFLP) Analysis

**Figure 4:**
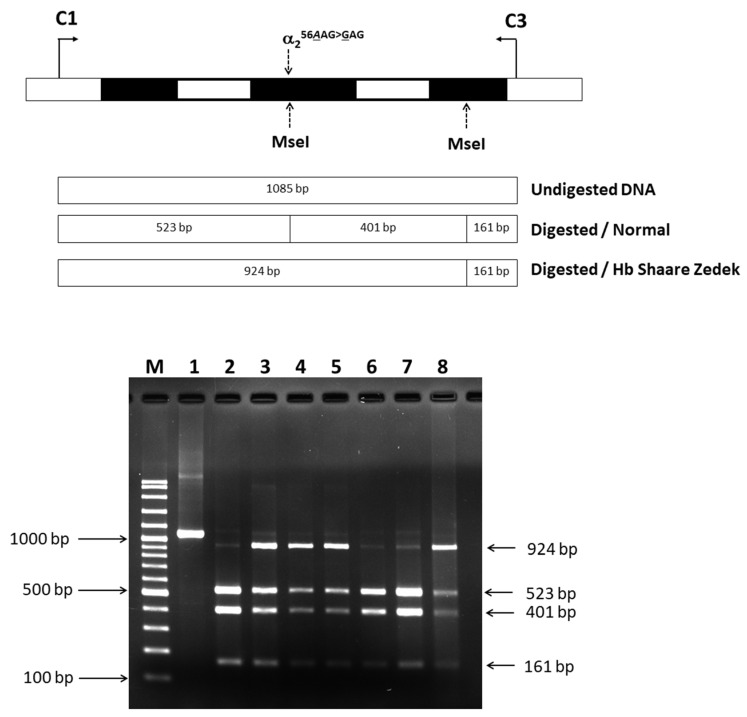
Identification of the AAG to GAG mutation using MseI RFLP analysis. Lane 1 shows the undigested PCR product. Lanes 2–8 show the digested products. Lanes 2, 6, and 7 represent MseI-digested PCR products from samples without the Hb SZ mutation (523, 401, and 161 bp). Lanes 3 (grandfather), 4 (father), 5 (proband), and 8 (aunt-1) show the presence of the 924 bp fragment specific to the Hb SZ allele.

The AAG to GAG mutation at codon 56 resulted in the loss of the MseI restriction site on the α2-globin gene. PCR amplification followed by MseI digestion of the amplified DNA fragments yielded distinct patterns for normal and Hb SZ alleles. Digestion of normal alleles produced fragments of 523, 401, and 161 bp, while a larger 924 bp fragment specific to Hb SZ was observed in the proband, father, grandfather, and aunt-1 (Figure 4). This confirmed the presence of the α^SZ^ allele in these individuals, verifying heterozygosity for Hb SZ.

### 2.4. Identification of Mutated Hb Variant via Allele-Specific PCR

**Figure 5:**
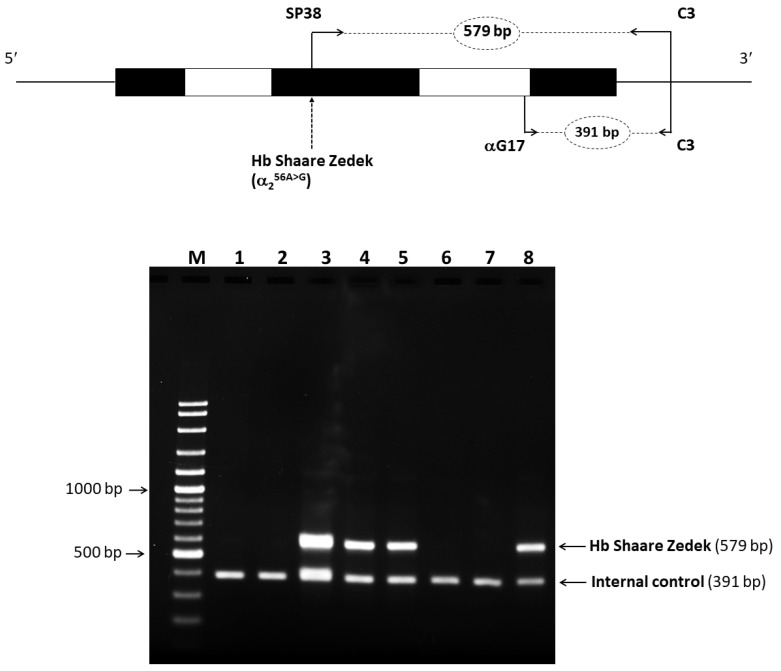
Allele-specific PCR for Hb SZ detection. Agarose gel electrophoresis of amplified fragments identified. A 579 bp band specific to the Hb SZ mutant allele, along with an internal control band at 391 bp in all lanes. Lane 1 represents the negative control, and positive results for the Hb SZ mutation are found in lanes 3 (grandfather), 4 (father), 5 (proband), and 8 (aunt-1).

We developed a highly efficient and reliable allele-specific PCR method for detecting the Hb SZ mutation, validated by DNA sequencing. In the proband and three family members carrying this variant, a distinct 579-bp fragment specific to the Hb SZ allele was detected. This fragment was absent in individuals without the mutation, demonstrating the success of our method. Additionally, a 391-bp fragment specific to the normal allele was identified (Figure 5). This method can significantly streamline and expedite the diagnosis of Hb SZ.

### 2.5. In Silico Analysis of Pathogenicity and Structure

**Figure 6:**
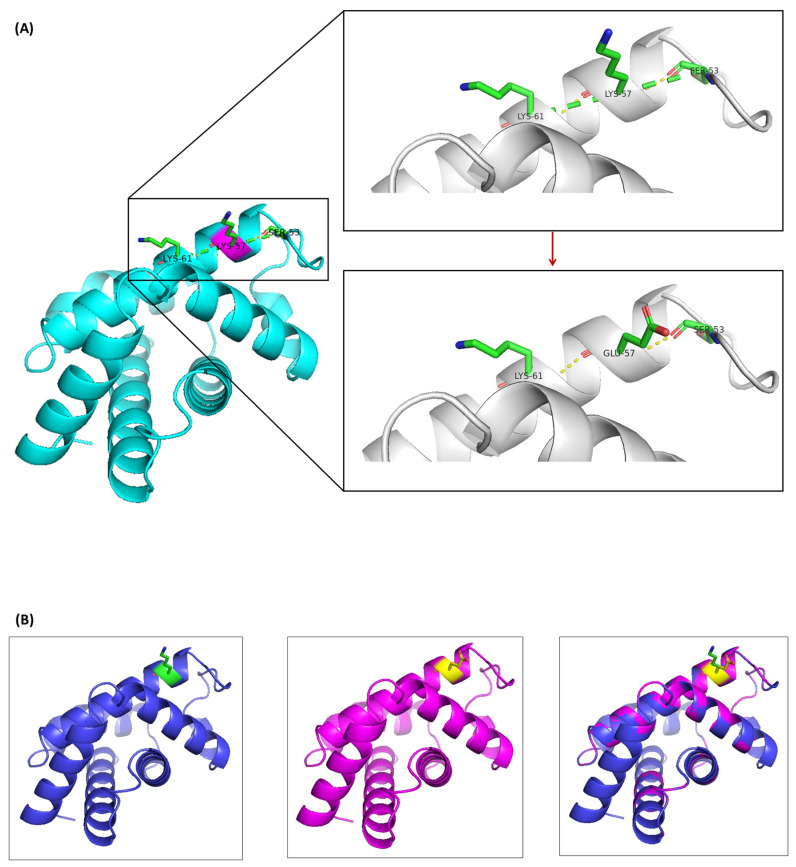
Structural analysis of the Hb SZ mutation. (**A**) Close-up view of the amino acid change in the α-globin chain caused by the Hb SZ mutation. The mutated Glu56 is connected with the side chains of Ser52 and Lys60. (**B**) Molecular model of deoxyhemoglobin showing the α-globin chain in a ribbon structure. **Left**: Normal α-globin protein with Lys56(E5) in green. **Center**: Predicted structure of the mutant α-globin chain in Hb SZ, with Glu56(E5) in yellow. **Right**: Superposition of the predicted mutant structure (pink) on the normal α-globin structure (blue).

PolyPhen-2 models, a computational tool for predicting pathogenicity, classified the Hb SZ mutation as likely benign with a score of 0.241 (sensitivity 0.91 and specificity 0.88). This prediction was supported by a molecular model constructed using SWISS-MODEL (https://swissmodel.expasy.org/ accessed on 31 May 2024) and visualized with PyMOL software (Version 3.0.3) (Figure 6). In the native HBA2 protein, lysine at position 28 (Lys28) is located on a α-helix forming hydrogen bonds with Ser52 and Lys60 (Figure 6A). Importantly, PyMOL analysis revealed that substituting Lys28 with glutamic acid did not result in significant structural changes in the predicted mutant Hb tetramer compared to the native form (Figure 6B). These findings suggest that the Hb SZ mutation did not significantly alter the tertiary structure of the Hb tetramer.

### 2.6. α-Globin Gene Haplotype Analysis

Alpha-globin gene haplotype analysis was performed to determine the specific combination of genetic variations inherited on a single chromosome. This analysis typically involves identifying the presence or absence of specific restriction enzyme cleavage sites within the α-globin gene cluster. The results are summarized in Table 2. The Hb SZ allele was significantly associated with the [- - M - - - -] haplotype.

## 3. Discussion

In this study, we presented Hb SZ in heterozygotes and in combination with β^0^-thalassemia, a novel combination first identified in a Thai family. The Thai Hb SZ mutation was discovered on the α2-globin gene, consistent with the findings in Israeli and Chinese populations [10,12].

An analysis of the amino acid substitution in the α-globin chain revealed that the mutation (lysine to glutamic acid at position 56) is located on the outer surface of the E segment of the α-helix. This residue does not participate in subunit interactions or the heme pocket, which are essential for hemoglobin function [13]. Structural analysis showed that the protein structure and the neighboring residues remained intact despite the mutation, preserving normal biological functions. Consequently, the replacement of lysine with glutamic acid at this position does not affect the protein’s function or stability. Hematological analysis revealed no anemia in the heterozygous carriers of this mutation, providing robust evidence of the functional similarity between Hb SZ and HbA. Additionally, prediction software confirmed the mutation to be benign. Therefore, individuals heterozygous for Hb SZ exhibit no clinical symptoms and normal red blood cell parameters.

While individuals with Hb SZ alone displayed normal red blood cell parameters, the combination of Hb SZ with β^0^-thalassemia resulted in microcytes and hypochromia (Figure S1). These changes are significantly influenced by the β^0^-thalassemia gene [14]. Other mutations at the same position as Hb SZ, including Hb Thailand (Lys>Thr HBA2:c.170A>C [or HBA1]), Hb Port Huron (Lys>Arg HBA2:c.170A>G [or HBA1]), and Hb Belliard (Lys>Asn HBA2:c.[171G>T {or HBA1} or 171G>C {or HBA1}]), have been documented [15,16,17]. Clinical cases involving these mutations show no abnormal hematological or clinical features. This further underscores our main finding that mutations at position 56 of the α2-globin chain do not significantly impact Hb stability or function and are not clinically significant.

The separation of Hb fractions is based on their electric charge in CE. Replacing the positively charged lysine with a negatively charged glutamic acid in Hb SZ caused the hemoglobin to have a net increase of two negative charges. As a result, the electrophoretic properties of Hb SZ differed from those of normal HbA. Hb SZ migrates faster than HbA, allowing it to be clearly separated from HbA. However, Hb SZ migrated to the position of zone 15, resembling the migration patterns of HbH and HbI (α16 [A14] Lys>Glu) [18,19]. Furthermore, in HPLC based on cation exchange (Premier Resolution HPLC), Hb SZ eluted faster than HbA. Unexpectedly, on the VARIANT II-HPLC, Hb SZ eluted at the same time as HbF, causing it to disappear from the chromatogram. These similar migration patterns with HbH or HbF make it challenging to estimate Hb SZ accurately using both CE and VARIANT II-HPLC. Fortunately, Premier Resolution HPLC can effectively differentiate Hb SZ from other Hb types, allowing for an accurate quantification in cases where other methods might be inconclusive.

This study is the first to reveal that Hb SZ constitutes approximately 23.5–25.0% of total Hb in simple heterozygotes, consistent with the amounts of other silent α-chain variants resulting from mutations on the α2-gene [20]. Notably, the level of Hb SZ in the proband, who displayed a co-inheritance of β^0^-thalassemia in a heterozygous Hb SZ, was significantly lower than those with a simple heterozygous Hb SZ (15.4%). This suggests that when the availability of the β-chain is reduced in β^0^-thalassemia, the formation of HbA (α_2_β_2_) is favored over the formation of Hb SZ (α^SZ^_2_β_2_).

Additionally, the HbA2 level quantified by CE in heterozygotes was significantly decreased, whereas the HPLC method detected it within the normal range [21]. Therefore, it is unsurprising that Hb analysis using CE can be misleading in diagnosing HbH disease. This is because the HbH fraction observed together with significantly low HbA_2_ levels is a characteristic feature found in individuals with the HbH disease [22,23]. Similarly, HPLC using the VARIANT II system can conceal Hb SZ within the HbF peak, potentially resulting in a misdiagnosis of β-thalassemia, even when HbA2 levels are within the normal range. This study underscores the importance of considering hematological characteristics in samples with abnormal Hb levels, particularly when Hb variants might be concealed within HbH or HbF peaks. This highlights the need for alternative techniques that can effectively separate different Hb species. Accurate diagnosis of Hb variants often requires molecular analysis. The allele-specific PCR method we developed offers a rapid and reliable tool to confirm this variant. This study represents the first report of Hb SZ with various genotypes in Southeast Asia. With improved diagnostic methods, it is likely that more such cases will be identified in the future.

The haplotype of the α-globin gene associated with Hb SZ in Thailand has been characterized for the first time in this study. The Thai Hb SZ mutation was found to be strongly linked to the haplotype [- - M - - - -]. This discovery enhances our understanding of the origins and evolution of the Hb SZ mutation in Thai populations. Since the α-globin gene haplotype of similar variants has not been previously reported, it remains unclear if the Thai Hb SZ shares the same ancestral origin as those reported in other populations. However, our research suggests a likely origin of the α-haplotype of Thai Hb SZ in Southeast Asia, marking a significant step forward in our understanding of this specific mutation.

In conclusion, our study sheds light on the clinical characteristics of Hb SZ and its impact on red blood cells. We found that Hb SZ, by itself, is a non-pathological hemoglobinopathy that does not significantly affect red cell parameters or clinical presentations. Even when combined with β^0^-thalassemia, the effects on red blood cell parameters are minimal. Traditional methods such as CE and standard HPLC cannot differentiate Hb SZ from other Hb, potentially leading to the misdiagnosis of hemoglobinopathies. Premier Resolution HPLC enables precise identification and quantification of Hb SZ, demonstrating its superior capability to resolve this variant. The identification of Hb SZ in this study has expanded the known spectrum of abnormal Hb found in the Thai population. This discovery provides valuable insights for future clinical diagnoses and genetic counseling related to hemoglobinopathies.

## 4. Materials and Methods

### 4.1. Study Participants and Hematological Analysis

We conducted a study involving a 5-year-old Thai boy with no history of organomegaly, icteric sclera, or blood transfusion, as well as six of his family members. Peripheral blood samples (2.5 mL) were collected using K_2_EDTA anticoagulant, which were analyzed for hematological parameters with a UniCel DxH 800 automated blood cell counter (Beckman Coulter Co., Brea, CA, USA). Hemoglobin analysis was performed using two automated cation-exchange HPLC systems: the Premier Resolution system (Trinity Biotech, in Bray, Country Wicklow, Ireland) in high-resolution mode and the VARIANT II system (Bio-Rad, Hercules, CA, USA) with the β-Thalassemia Short Program. Additionally, CE was conducted using the MINICAP Flex Piercing system (Sebia, Lisses, France).

To identify HbH inclusion bodies (excess β chains assembled into tetramers; β4), we used brilliant cresyl blue as an oxidant. It denatures intracellular HbH into intracellular inclusions. Peripheral blood smears were air-dried and mixed with 1% BCB at a ratio of one-part BCB to one-part blood. The mixture was incubated at 37 °C for 1 h, and the inclusions formed were examined under an oil immersion objective typically assessing 5000 red blood cells for the presence of inclusions [24]. This study protocol was approved by the Ethics Committee and Institutional Review Board of the University of Phayao in Phayao, Thailand (approval no. 1.2/026/67). All participants provided written informed consent before participating in this study.

### 4.2. Molecular Characterization

Genomic DNA was extracted from the white blood cells isolated from K_2_EDTA-treated blood using a commercially available genomic DNA isolation kit (Bio-Helix Co., LTD., in Keelung City, Taiwan). Mutations in the α1- and α2-globin genes were analyzed by PCR amplification using a previously described protocol [25]. The amplified genes were directly sequenced with an ABI PRISM^TM^ 3130 xl analyzer (Applied Biosystems, Foster City, CA, USA). Mutation positions were identified and analyzed using the human abnormal Hb thalassemia library (http://globin.bx.psu.edu (accessed on 24 July 2024)). Additionally, common α^0^-thalassemia deletions (--^SEA^ and --^THAI^), α^+^-thalassemia deletions (-α^3.7^ [rightward] and -α^4^.^2^ [leftward]), and the common Hb Constant Spring (HbCS; HBA2: c.427T>C) and Hb Paksé (HBA2: c.429A>T) were routinely screened using Gap-PCR and allele-specific PCR [26,27,28].

### 4.3. Identification of Hb SZ by PCR-RFLP

A substitution of A to G at the first nucleotide of codon 56 on the α2-globin gene results in the loss of an MseI recognition site. We developed a PCR-RFLP to confirm this mutation. Amplification of a 1085-bp fragment specific for the α2-globin gene was performed using primers C1 (5′-TGGAGGGTGGAGACGTCCTG-3′) and C3 (5′-CCATTGTTGGCACATTCCGG-3′). The PCR reaction mixture (50 μL) included genomic DNA, 60 pmol of primers C1 and C3, 200 μM deoxyribonucleotides (dNTPs), 1.5 mM MgCl_2_, 1 M betaine, 5% dimethyl sulfoxide (DMSO), and 1.5 units of *Taq* DNA polymerase (Vivantis Technologies, Selangor Darul Ehsan, Malaysia) in a buffer containing 10 mM Tris-HCl (pH 9.1), 50 mM KCl, and 0.1% Triton^TM^ X-100. Amplification was conducted on a thermal cycler (Cyclerus personalis, Bio-Rad, USA) with an initial denaturation at 94 °C for 3 min, followed by 10 cycles of 94 °C for 30 s, 60 °C for 30 s, and 68 °C for 2 min. This was followed by 20 cycles of 94 °C for 30 s, 60 °C for 30 s, and 68 °C for 2 min, with an additional 20 s per cycle. The amplified fragment was then digested with the MseI restriction enzyme (5′-TT^▼^AA-3′) (New England Biolabs, Beverly, MA, USA) and analyzed by 2.0% agarose gel electrophoresis. The digested fragments were visualized under UV light after ethidium bromide staining. MseI digestion of a normal allele produced 523 bp, 401 bp, and 161 bp fragments, whereas the Hb SZ allele resulted in 924 bp and 161 bp fragments.

### 4.4. Allele-Specific PCR for Rapid Identification of Hb SZ

We developed an efficient method for diagnosing Hb SZ using allele-specific PCR. A specific forward primer, SP38 (5′-CACGGCTCTGCCCAGGTTG-3′), located within exon 1 of the α2-globin gene and specific to the A>G mutation, was designed. This primer, along with the previously mentioned reverse primer C3, produces a 579-bp fragment specific to the α^SZ^ allele. Additionally, primers αG17 (5′-AGATGGCGCCTTCCTCTCAGG-3′) and C3 were used to generate a 391-bp fragment as an internal control.

The total PCR volume (50 μL) included genomic DNA, 30 pmol of primers SP38 and C3, 7.5 pmol of primer αG17, 200 μM dNTPs, 1.5 mM MgCl_2_, 5% DMSO, 1 M betaine, and 1.5 units of *Taq* DNA polymerase in a buffer containing 10 mM Tris-HCl (pH 9.1), 50 mM KCl, and 0.1% Triton^TM^ X100. Amplification was conducted on a thermal cycler (Cyclerus personalis, Bio-Rad, USA) with an initial denaturation step at 95 °C for 15 min, followed by 2 cycles of 95 °C for 60 s, 65 °C for 60 s, and 72 °C for 90 s; then 30 cycles of 95 °C for 60 s, 65 °C for 30 s, and 72 °C for 90 s; and a final extension at 72 °C for 10 min. The amplified product was analyzed by electrophoresis on 1.5% agarose gel and visualized under UV light after ethidium bromide staining.

### 4.5. Analysis of α-Globin Gene Haplotypes

α-globin gene haplotypes for all participants were identified using seven common polymorphisms. These included six RFLPs and one broadly triallelic inter-ζ-globin hypervariable region (HVR). The RFLPs targeted specific sites: the *Xba*I site of the 5′ ζ2-globin gene, the *Bgl*I site of the 3′ ζ2-globin gene, the *Acc*I site of the 3′ ψα2-globin gene, the *Rsa*I site of the 5′ α2-globin gene, and the *Pst*I sites of the 5′ α1- and 5′ θ1-globin genes. Amplification of these regions was based on established and reproducible methodologies [29]. Haplotypes were determined by analyzing the presence or absence of cleavage at each site and combining these outcomes into a unified pattern.

### 4.6. In Silico Modeling of Hb SZ

To better understand the impact of amino acid substitution on the structure and function of the α-globin chain, predictions were made using the HumDiv-trained network-based analysis tool, Poly Phen-2 (http://genetics.bwh.harvard.edu/pph2/ (accessed on 24 July 2024)). Additionally, molecular modeling of normal and variant proteins was conducted using SWISS-MODEL (https://swissmodel.expasy.org/ (accessed on 24 July 2024)). Visualization and comparative analysis of the 3D protein structures were carried out using the PyMOL Molecular Graphics System (Version 3.0.3, Schrödinger, New York, NY, USA).

## Figures and Tables

**Table 1:** Hematological and genetic analyses of the proband and his family members.

Parameters	Grandfather	Grandmother	Paternal Aunt-1	Paternal Aunt-2	Father	Mother	Proband
Age (year)	65	59	34	31	36	37	5
RBC (×10^12^/L)	4.77	4.60	4.14	4.71	5.52	5.59	5.75
Hb (g/dL)	14.4	13.6	11.5	13.7	15.4	11.9	10.3
Hct (%)	44.3	43.2	36.3	42.5	48.3	34.7	31.2
MCV (fL)	92.8	94.1	87.5	90.2	87.5	62.1	54.3
MCH (pg)	30.3	29.6	27.8	29.1	27.9	21.3	17.9
MCHC (g/dL)	32.6	31.5	31.7	32.3	31.9	34.3	33
RDW-CV (%)	13.4	12.7	13.0	13.5	14.0	16.6	18.9
CE-Hb profile ^a^	A2AH	A2A	A2AH	A2A	A2AH	A2A	A2AH
Hb A (%)	67.5	67.5	66.5	97.1	67.6	93.8	81.5
Hb A2 (%)	1.9	2.9	1.8	2.9	1.9	5.8	5.3
Hb F (%)	0	0	0	0	0	0.4	0
Hb SZ (%)	30.6	0	31.7	0	30.5	0	13.2
Premier HPLC-Hb Profile ^b^	A2A with Hb SZ	A2A	A2A with Hb SZ	A2A	A2A with Hb SZ	A2A	A2A with Hb SZ
Hb A (%)	66.2	86.9	66.7	88.6	64.7	86.8	70.6
Hb A2 (%)	2.3	3.2	2.3	3.2	2.5	5.9	5.4
Hb F (%)	0	0	0	0	0	1.1	1.3
Hb SZ (%)	23.5	0	25.0	0	24.7	0	15.4
VARIANT II HPLC-Hb Profile ^c^	A2FA	ND	A2FA	ND	A2FA	ND	A2FA
Hb A (%)	64.4	ND	64.4	ND	66.2	ND	70.9
Hb A2 (%)	2.3	ND	2.3	ND	2.5	ND	5.3
Hb F + Hb SZ	18.3	ND	19.5	ND	22.6	ND	14.0
α-globin genotype	α^56A>G^α/αα	αα/αα	α^56A>G^α/αα	αα/αα	α^56A>G^α/αα	αα/αα	α^56A>G^α/αα
β-globin genotype	β^A^/β^A^	β^A^/β^A^	β^A^/β^A^	β^A^/β^A^	β^A^/β^A^	β^A^/β^0^	β^A^/β^0^

RBC, red blood cell; Hb, hemoglobin; Hct, hematocrit; MCV, mean corpuscular volume; MCH, mean corpuscular hemoglobin; MCHC, mean corpuscular hemoglobin concentration; RDW-CV, coefficient of variation of the red cell distribution width; Hb SZ, Hb Shaare Zedek. ^a^ Determined using capillary electrophoresis (CE), Capillary 2. ^b^ Determined using high-performance liquid chromatography (HPLC), Premier Resolution, Trinity Biotech. ^c^ Determined using HPLC, Bio-Rad VARIANT II system.

**Table 2:** α-globin gene haplotypes associated with Hb SZ.

α-Globin Haplotype (5′>3′)																
Subjects	α-Genotype	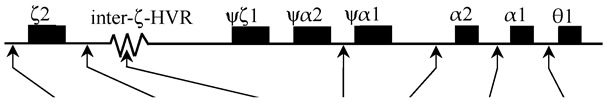							Hb SZ (α ^SZ^) Linked α-Haplotype							
		*Xba* I	*Sac* I	[S/M/L]	*Acc* I	*Rsa* I	α*Pst* I	θ*Pst* I								
Grandfather	αα ^SZ^/αα	[+/−]	[+/−]	[M/M]	[+/−]	[−/−]	[+/−]	[−/−]	**αα ^SZ^**	**[-**	**-**	**M**	**-**	**-**	**-**	**-]**
									αα	[+	+	M	+	-	+	-]
Grandmother	αα/αα	[+/−]	[+/−]	[L/S]	[+/+]	[+/−]	[−/−]	[+/−]	αα	[+	-	S	+	+	-	+]
									αα	[-	+	L	+	-	-	-]
Paternal Aunt-1	αα ^SZ^/αα	[+/−]	[−/−]	[M/S]	[+/−]	[+/−]	[−/−]	[+/−]	**αα ^SZ^**	**[-**	**-**	**M**	**-**	**-**	**-**	**-]**
									αα	[+	-	S	+	+	-	+]
Paternal Aunt-2	αα/αα	[+/−]	[+/+]	[L/M]	[+/+]	[−/−]	[+/−]	[−/−]	αα	[-	+	L	+	-	-	-]
									αα	[+	+	M	+	-	+	-]
Father	αα ^SZ^/αα	[−/−]	[+/−]	[L/M]	[+/−]	[−/−]	[−/−]	[−/−]	**αα ^SZ^**	**[-**	**-**	**M**	**-**	**-**	**-**	**-]**
									αα	[-	+	L	+	-	-	-]
Mother	αα/αα	[+/−]	[+/−]	[L/S]	[+/+]	[−/−]	[+/−]	[+/−]	αα	[+	-	S	+	-	+	+]
									αα	[-	+	L	+	-	-	-]
Proband	αα ^SZ^/αα	[+/−]	[−/−]	[M/S]	[+/−]	[−/−]	[+/−]	[+/−]	**αα ^SZ^**	**[-**	**-**	**M**	**-**	**-**	**-**	**-]**
									αα	[+	-	S	+	-	+	+]

+ and − represent the presence and absence of the restriction enzyme sites, respectively. S and M indicate inter-ζ hyper-variable region (HVR).

## Data Availability

Data is contained within the article.

## Supplementary Materials

The following supporting information can be downloaded at: https://www.mdpi.com/article/10.3390/ijms25168578/s1.
