# Supplementary material for: Novel Insights into Hb Shaare Zedek Associated with β0-Thalassemia: Molecular Characteristics, Genetic Origin and Diagnostic Approaches
Source: Int J Mol Sci. 2024 Aug 6;25(16):8578. doi: 10.3390/ijms25168578 (PMC11354257; doi:10.3390/ijms25168578)
Supplement: Supplementary file 1 [file ijms-25-08578-s001.zip › ijms-3092458-supplementary.pdf]

Supplementary information

(A)

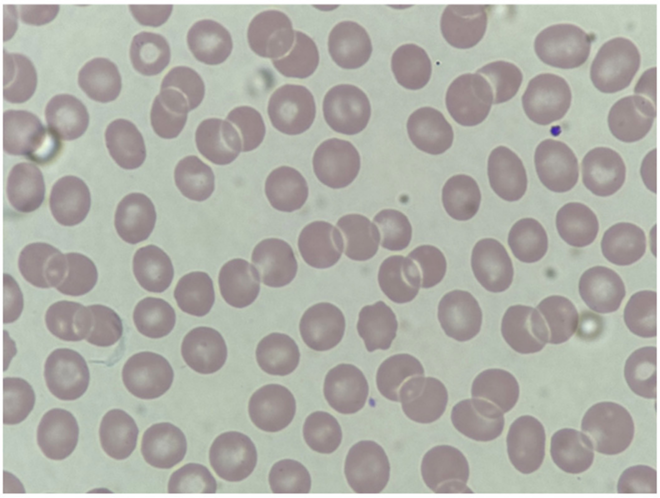

(B)

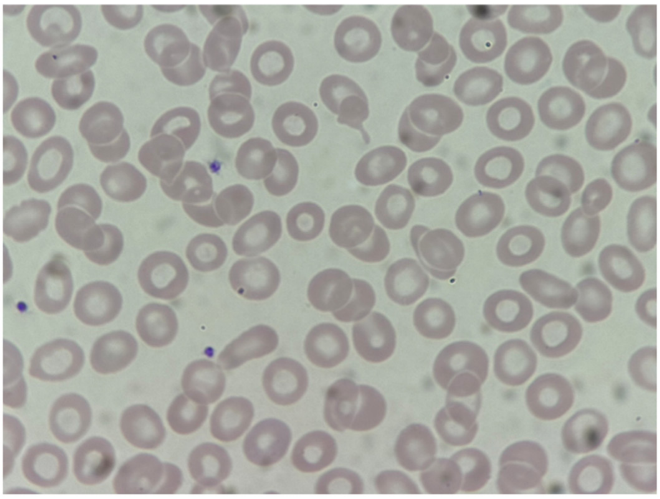

(C)

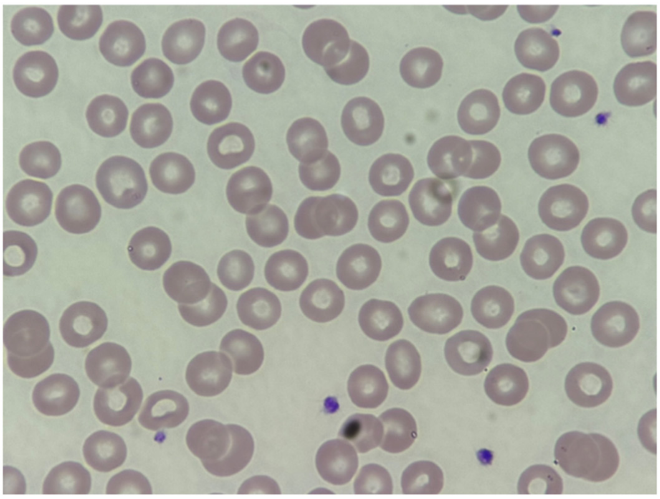

Figure S1. Wright-Giemsa staining of peripheral blood smears from family members.

(A) Peripheral blood smear of the father.

(B) Peripheral blood smear of the proband.

(C) Peripheral blood smear of the mother.
